# Characteristics of primary care practices associated with patient education during COVID-19: results of the cross-sectional PRICOV-19 study in 38 countries

**DOI:** 10.1186/s12875-024-02348-x

**Published:** 2024-04-18

**Authors:** Delphine Kirkove, Sara Willems, Esther Van Poel, Nadia Dardenne, Anne-Françoise Donneau, Elodie Perrin, Cécile Ponsar, Christian Mallen, Neophytos Stylianou, Claire Collins, Rémi Gagnayre, Benoit Pétré

**Affiliations:** 1 Department of Public Health Sciences, University of Liege, B23 / Avenue Hippocrate, n°13, 4000 Liège, Belgium; 2 Biostatistics and Research Method Center, University of Liege, Liege, Belgium; 3 Department of Public Health and Primary Care, Ghent University, Ghent, Belgium; 4 Quality and Safety Ghent, Department of Public Health and Primary Care, Ghent University, Ghent, Belgium; 5 Institute of Health and Society, Louvain University, Louvain, Belgium; 6 School of Medicine, University of Keele, Keele, UK; 7 International Institute for Compassionate Care, Nicosia, Cyprus; 8 NS Intelligence Solutions Ltd, Nicosia, Cyprus; 9 Education and Health Practices Laboratory (LEPS), (UR 3412), UFR SMBH, Sorbonne Paris-Nord University, Bobigny, France; 10 Research Centre, Irish College of General Practitioners, Dublin, D02XR68 Ireland

**Keywords:** Primary health care, General practice, Patient education, PRICOV-19, COVID-19, International comparison

## Abstract

**Background:**

In response to the COVID-19 pandemic, the World Health Organization established a number of key recommendations such as educational activities especially within primary care practices (PCPs) which are a key component of this strategy.

This paper aims to examine the educational activities of PCPs during COVID-19 pandemic and to identify the factors associated with these practices across 38 countries.

**Methods:**

A "Patient Education (PE)" score was created based on responses to six items from the self-reported questionnaire among PCPs (*n* = 3638) compiled by the PRICOV-19 study. Statistical analyses were performed on 3638 cases, with PCPs with missing PE score values were excluded.

**Results:**

The PE score features a mean of 2.55 (*SD* = 0.68) and a median of 2.50 (2.16 – 3.00), with a maximum of 4.00, and varies quite widely between countries. Among all PCPs characteristics, these factors significantly increase the PE score: the payment system type (with a capitation payment system or another system compared to the fee for service), the perception of average PCP with patients with chronic conditions and the perception of adequate governmental support.

**Conclusion:**

The model presented in this article is still incomplete and requires further investigation to identify other configuration elements favorable to educational activities. However, the results already highlight certain levers that will enable the development of this educational approach appropriate to primary care.

**Supplementary Information:**

The online version contains supplementary material available at 10.1186/s12875-024-02348-x.

## Background

The COVID-19 pandemic has had a major impact on health systems worldwide and has placed significant pressure on them [1]. Although national contexts differ, most countries have adopted similar policy responses to minimize the spread of the disease and its impact on the morbidity and mortality of their populations, such as stay at home lockdowns, closure of schools, … [2, 3]. Preventive strategies are central to the measures implemented, as they aim to avoid subsequent overload of health services. In addition to vaccination, the most common measures universally adopted by countries were wearing masks, regular hand washing and sanitization and physical distancing [4]. These measures aim to "*improve public health by identifying malleable risk and protective factors, assessing the efficacy and effectiveness of preventive interventions, and identifying optimal means for dissemination and diffusion*" [5] (page 36).

One component of preventative strategies is the educational approach. Regardless the target audience (e.g. general population or patients with chronic diseases), education is considered therapeutic because many studies have shown the positive effect of therapeutic patient education (TPE) on individual health [6]. TPE was defined in 1998 by the WHO as "*educational activities (…), designed to help a patient (…) to manage their treatment and prevent avoidable complications, and to maintain or improve their quality of life*" [7]. Some countries, such as France, Germany, Poland and Romania, have made "explicit recommendations" during the COVID-19 pandemic regarding patient education, such as the importance of personal hygiene or home care [8].

Although COVID-19 pandemic affects the whole population, certain populations are at higher risk, either because of their health status or because of difficulties in implementing prevention measures As early as April 2020, the High Council for Public Health in France confirmed the risk of severe forms of COVID-19 in people with co-morbidities [9]. These patients are not only a population at risk, but they have suffered even more from containment measures. These measures have severely curtailed the interactions with health professionals, affecting the continuity of care [10]. For example, a survey conducted in the first wave of the COVID-19 pandemic reported that almost 50% of Belgians forewent health care during this period [11]. Another study in France in 2020 demonstrated the same results [12]. This renunciation of care is not without consequences, as various studies indicate that it is often associated with a deterioration in health status [13].

Another vulnerability factor is health literacy, a skill that is particularly important to be able in using health information. Sorensen et al. define this concept as "*the knowledge, motivation and skills needed to access, to understand, evaluate and apply health information in order to make judgments and decisions in everyday life about health care, disease prevention and health promotion to or improve the quality of life throughout the life span*" [14]. Although there is some variability between European countries, the study of Sorensen et al. in the pre-COVID era showed that "*almost one in two people (47%) had limited (insufficient or problematic) health literacy”* [14]. While health literacy would help people better integrate and position themselves in relation to information and recommendations on COVID-19, it remains "*an underestimated public health problem worldwide*" [15]. Therefore, preventive health actions are difficult to implement in populations with lower health literacy, making them even more vulnerable during the COVID-19 pandemic [16].

Dealing with these different types of vulnerabilities involves not only individual skills, but also collective responsibility. They imply a community approach and actions to be taken on the environment in order to make it "*more enabling*" [17]. These elements constitute a real challenge for health care structures and the health system [16].

Primary care (PC) plays a key role especially regarding educational aspects of health because it is often the first point of contact for the general population. In a 2015 report, WHO Europe and WONCA Europe stated that PC should "*promote health and well-being through prevention and therapeutic education*" [18]. During the COVID-19 pandemic, it was expected that this educational role would be strengthened as PC professionals were the first point of contact for most COVID-patients with mild symptoms and responsible for short- and long-term follow-up care. In addition, they were also key figures for regular care for the majority of the population, providing access to hospital care as a gatekeeper, for example [19]. However, PC professionals faced many challenge in fulfilling their roles during COVID-19 pandemic in terms of coordination, continuity of care, comprehensiveness and/or accessibility. The context of uncertainty that has characterized this period has further amplified these difficulties [19]. It is therefore important to explore the practices of professionals in PC, as well as the adaptations they made to cope with the COVID-19 pandemic. While much research was conducted during the COVID-19 pandemic, it focused mainly on hospital care in the first months of the health crisis. Even in the field of patient education, the research has focused on the issue of continuity of care and the adaptation of educational programs [17, 20, 21], leaving PC relatively unexplored [19].

The aims of this paper were two-fold: (i) to describe the educational activities undertaken by primary care practices (PCPs) during the COVID-19 pandemic, and (ii) to examine the factors on practice and country level, which have influenced these educational activities.

## Materials and methods

### Study design and setting

In the summer of 2020, an international consortium of more than 45 research institutes was formed under the coordination of Ghent University (Belgium) to investigate how PCPs were organized during the COVID-19 pandemic (PRICOV-19). This multi-country cross-sectional study aims to analyze how PCPs worked during COVID-19 pandemic to guarantee high-quality care; how the task roles changed; how the COVID-19 pandemic impacted the well-being of care providers; and whether differences could be found between types of practices and/or healthcare systems. PRICOV-19 also aims to study the association with practice and health care system characteristics. Data were collected in 38 countries in Europe and in Israel. The study protocol and data handling protocols are described in the Data Management Plan registered at Ghent University [19].

Data were collected by means of an online self-reported questionnaire among PCPs. The questionnaire was developed at Ghent University in multiple phases, including a pilot study among 159 general practices in Flanders (Belgium). More details are described elsewhere [19].

The questionnaire was translated into 38 languages following a standard procedure. The Research Electronic Data Capture (REDCap) platform was used to host the questionnaire in all languages, to send out invitations to the national samples of PCPs, and to securely store the answers from the participant [22].

### Sampling and recruitment

Data were collected between November 2020 and December 2021, except for Belgium, where data was partially collected earlier. Data collection varied between countries from three to 35 weeks. In each partner country, the consortium partner(s) recruited PCPs following a pre-defined recruitment procedure. Drawing a randomized sample among all PCPs in the country was preferred over convenience sampling. Partners logged all the steps taken in the sampling procedure.

PRICOV-19 aimed to sample between 80 and 200 PCPs per country, depending on the number of GP practices. One questionnaire was completed per practice, preferably by a GP or by a staff member familiar with the practice organization. The overall response rate across all countries was 22%, ranging from 1.55% in Denmark to 94.3% in Bulgaria.

### Data analysis

Ghent University was responsible for the data cleaning. For this paper, a score for educational activities, entitled "Patient Education (PE)", was created based on the following six items from the PRICOV-19 self-reported questionnaire [19]:

- The involvement of staff members in providing information to patients by telephone
- The involvement of staff members in providing information to patients with low literacy skills
- The involvement of staff members in providing information to patients postponing their health care
- The involvement of general practitioners (GPs) or GP trainee’s in providing information to patients postponing their health care
- Checking with the patient that it is feasible to isolate themselves at home
- The mobilization by staff of a leaflet on COVID-19 to inform patients

The responses to these five items are all presented in the form of a 5-level Likert scale: level 0 corresponds to "*strongly disagree*" and level 4 to "*strongly agree*" for the first four items, and for the fourth item, level 0 corresponds to "*never*" and level 4 to "*always*". The last item on COVID-leaflets is also presented as a Likert scale, but with three levels and has been extrapolated using the rule of three to a 5-level scale. In the end, each item has a score between 0 and 4. Then, an average was calculated based on these six items to constitute the PE score.

For the characteristics of the PCPs, eight indicators were used: the number of GPs per practice, the main payment system, the perception of the PCP in terms of the proportion of patients with limited health knowledge or low literacy, or patients with chronic conditions in their practice compared to the average in their country, the number of patients registered in the practice, the multidisciplinarity of practice team, administrative support in practice and the perception of adequate government support. The first indicator refers to the number of GPs per practice categorized into three groups: “solo”, “duo” and “group” (i.e. at least three GPs working in practice) irrespective of being part/full time. The variable “Main payment system” was *a “centrally created variable merging the relevant response options in each country into three overall categories appropriate for international comparison”* (i.e., fee-for-service, capitation, other and/or mixed) [23]. The “multidisciplinary team” variable includes a range of professionals working in PC, other than the GP: social worker, psychologist, dietitian or nutritionist, physiotherapist or manual therapist, podologist and nurse or nurse assistant. The “administrative support” variable reflects professionals working in PC, excluding the GP and the category “multidisciplinary support”: receptionist, administrative assistant or practice manager. The variable of “the perception of an adequate government support for the practice functioning” variable was categorized as “*(strongly) disagree*”, “*neutral*” and “*(strongly) agree*”. The explanatory variables were selected based on the literature as well as on initial data mining.

PCPs with missing 'PE score' data were excluded (*n* = 5320), so analyses were performed on 3638 cases.

Data are summarized using frequency and percentage for categorical parameters, and mean and standard deviation according to the normality of the distribution of the variable or median and interquartile range (P25—P75) for continuous parameters (not normally distributed).

Linear mixed model analysis (LMM) were performed (due to the clustering of respondent practices in countries), with the continuous PE score as the outcome variable. The conditions for linear mixed model analysis were checked (normality and homoscedasticity of residuals). Results are shown in the additional files 1 and 2. We tested different random intercept models using restricted maximum likelihood estimation. Three models were tested; Model 1 with significant covariates in univariate analyses as fixed effect; Model 2 with “the primary care practice with patients with limited low literacy” added as fixed effect; Model 3 with the number of GPs per practice added as fixed effect. The null model was also computed in order to calculate the intraclass correlation coefficient (ICC), wich assess the proportion of the variance in the outcome variable that can be explained by country. The covariance structure modelled was heterogeneous Toeplitz [24]. The Akaike’s Information Criterion (AIC), the Bayesian Information Criterion (BIC) and 2 log-likelihood values were used as goodness-of-fit model criteria. The likelihood ratio test was used to compare model fit between nested models.

Statistical analysis was performed using SAS (version 9.4) and R software (version 4.0.3). The results are considered significant at the 5% uncertainty level.

### Ethical approval

The study was conducted according to the guidelines of the Declaration of Helsinki. The Research Ethics Committee of Ghent University Hospital approved the protocol of the PRICOV-19 study and Belgian data collection (BC-07617). Research Ethics Committees in the different partner countries gave additional approval if needed. All participants gave informed consent on the first page of the online questionnaire.

## Results

A total of 3638 respondents (40.61% of the total) were included in the analysis based on complete answers to the questions used to calculate the PE score.

The characteristics of the sample are presented in Table 1. The majority of them had a professional group practice (50.67%) and a capitation payment system (45.15%). About half of the practices consider themselves to have a comparatively average number of patients with low or limited literacy and patients with a chronic condition when compared to other practices in their area. The number of patients registered in the practices varies from less than 1500 patients to more than 7000 per facility. The staff is diversified, with multidisciplinary and administrative support present in almost 70% of the facilities. More than half of the PCPs (52.20%) considered they did not receive adequate government support for the practice organization.

**Table 1:** Description of the characteristics of the sample (*n* = 3638)

	**n**	**%**
**Number of GPs per practice**		
Solo	1258	34.96
Duo	517	14.37
Group (≥ 3 GPs)	1823	50.67
**Main payment system**		
Fee-for-service	1246	34.92
Capitation	1611	45.15
Other and /or mixed	711	19.93
**Perception of average primary care practice with patients with limited health literacy or low literacy**		
Below average	1168	33.45
Approximately the average	1635	46.82
Above average	689	19.73
**Perception of average primary care practice with patients with chronic conditions**		
Below average	149	04.19
Approximately the average	1911	53.68
Above average	1500	42.13
**Number of patients registered in practice of GP**		
≤ 1500	523	14.77
> 1500—≤ 3000	1152	32.52
> 3000—≤ 7000	821	23.18
> 7000	1046	29.53
**Multidisciplinarity of the practice team**		
No	1019	28.11
Yes	2606	71.89
**Availability of administrative support**		
No	1346	37.11
Yes	2281	62.89
**Perception of adequate governmental Support**		
(Strongly) disagree	1845	52.22
Neutral	839	23.75
(Strongly) agree	849	24.03

**Fig. 1:**
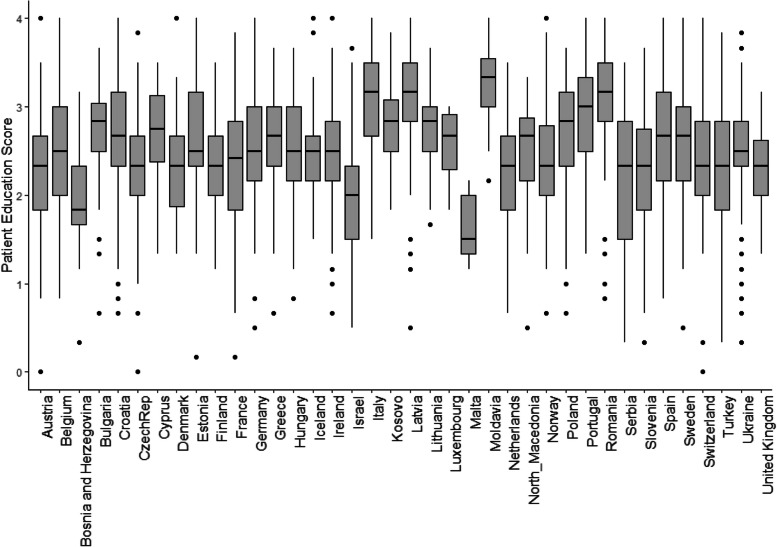
Box plot of PE score by country during the COVID-19 pandemic (*n* = 3638)

The PE score varies between 0 (min.) and 4 (max.), with 4 indicating a high score for educational activities during the COVID pandemic. The score features a mean of 2.55 (*SD* = 0.68) and a median of 2.50 (2.16 – 3.00). As shown in Fig. 1, the PE score varies quite widely between countries, with high dispersion also within countries.

Table 2 shows the results of the univariate analyses carried out between the continuous “PE score” and the characteristics of the practices. With the exception of two variables (number of GPs per practice and the perception of average primary care practice with patients with limited health literacy or low literacy), all the other variables are significantly associated with the PE score, given the Type 3 p value (under 5%).

**Table 2:** Results of univariate analysis (LMM) between the “PE score” and practice characteristics (*n* = 3638)

	**PE Score (*Mean* ± *SD*)**	***Coefficient* ± *SE***	**p value**	**Type 3 *p* value**
**Number of GPs per practice**				0.11
Solo	2.58 ± 0.69	/		
Duo	2.47 ± 0.69	0.007 ± 0.04	0.840	
Group (≥ 3 GPs)	2.54 ± 0.67	0.06 ± 0.03	0.056	
**Perception of average primary care practice with patients with limited health literacy or low literacy**				0.052
Below average	2.51 ± 0.71	/		
Approximately the average	2.55 ± 0.66	0.05 ± 0.02	0.035	
Above average	2.59 ± 0.68	0.07 ± 0.03	0.038	
**Perception of average primary care practice with patients with chronic conditions**				**0.011**
Below average	2.41 ± 0.73	/		
Approximately the average	2.52 ± 0.68	0.09 ± 0.05	0.009	
Above average	2.59 ± 0.67	0.14 ± 0.05	0.080	
**Main payment system**				**0.0075**
Fee-for-service	2.42 ± 0.66	/		
Capitation	2.60 ± 0.68	0.11 ± 0.04	0.005	
Other and /or mixed	2.62 ± 0.68	0.01 ± 0.05	0.011	
**Number of patients registered in practice of GP**				**0.028**
≤ 1500	2.46 ± 0.69	/		
> 1500—≤ 3000	2.52 ± 0.67	0.07 ± 0.03	0.053	
> 3000—≤ 7000	2.52 ± 0.67	0.06 ± 0.04	0.100	
> 7000	2.63 ± 0.68	0.12 ± 0.04	0.003	
**Multidisciplinarity of the practice team**				**0.0007**
No	2.46 ± 0.66	/		
**Yes**	2.57 ± 0.68	0.09 ± 0.03	0.0007	
**Availability of administrative support**				**0.017**
No	2.48 ± 0.70	/		
Yes	2.58 ± 0.67	0.06 ± 0.03	0.017	
**Perception of adequate governmental Support**				**< 0.0001**
(Strongly) disagree	2.50 ± 0.69	/		
Neutral	2.53 ± 0.66	0.07 ± 0.03	0.011	
(Strongly) agree	2.66 ± 0.70	0.17 ± 0.03	< 0.0001	

Regarding multivariate analysis, the results of the Linear mixed model with the continuous “PE Score” and the characteristics of primary care practices are shown in Table 3. Three models were tested:

- A “null” model was calculated and added to the multivariate model. The IntraClass Correlation (ICC) here is 17.2%, which means that 17% of the variance of the PE score is attributable to the country.
- Model I included significant covariates in univariate analyses as fixed effect and shows that three variables still remain significant: perception of average primary care practice with patients with chronic conditions (*p* = 0.0032), the multidisciplinary team (*p* = 0.032) where the "PE" score increases with the presence of a multidisciplinary team (Coef ± SE = 0.06 ± 0.03) and the agreement of the perception of adequate governmental support (*p* < 0.0001, Coef ± SE = 0.15 ± 0.03).
- Model II, with the addition of “Perception of average primary care practice with patients with limited low literacy” as fixed effect, shows the same results except for the multidisciplinary teams, non-significant in this model (*p* = 0.074).
- Based on goodness-of-fit criteria, the best model is model III, with the addition of the number of GPs per practice as fixed effect. Nevertheless, the difference in AIC values between model I (AIC = 6471.20) and model III (AIC = 6251.30) is not very high. The main payment system is only significant in this model (*p* = 0.047) where the "PE" score increases with a capitation payment system (Coef ± SE = 0.09 ± 0.04) or another system (Coef ± SE = 0.11 ± 0.05) compared to the fee for service. The perception of average primary care practice with patients with chronic conditions (*p* = 0.011, Coef ± SE = 0.14 ± 0.05) and the perception of an adequate governmental support (*p* < 0.0001, Coef ± SE = 0.17 ± 0.03) remain significant.

**Table 3:** Results of Multivariate (LMM) analysis (Continuous “PE score” during COVID 19)

	**Model Null**	**Model I**	**Model II**	**Model III**
	**Coefficient ± SE**	**Coefficient ± SE**	**Coefficient ± SE**	**Coefficient ± SE**
**Intercept**	2.52 ± 0.05 ***	2.15 ± 0.08 ***	2.16 ± 0.09 ***	2.14 ± 0.09 ***
**Number of GPs per practice**				
Solo				/
Duo				0.01 ± 0.04
Group				0.05 ± 0.04
**Perception of average primary care practice with patients with limited health literacy or low literacy**				
Below average			/	/
Approximately the average			0.04 ± 0.03	0.04 ± 0.03
Above average			0.05 ± 0.03	0.05 ± 0.03
**Perception of average primary care practice with patients with chronic conditions**				
Below average		/	/	/
Approximately the average		0.16 ± 0.06	0.08 ± 0.06	0.08 ± 0.06
Above average		0.11 ± 0.06 **	0.13 ± 0.06 *	0.14 ± 0.06 *
**Main payment system**				
Fee-for-service		/	/	/
Capitation		0.08 ± 0.04	0.09 ± 0.04	0.09 ± 0.04 *
Other and /or mixed		0.11 ± 0.05	0.10 ± 0.06	0.11 ± 0.05*
**Number of patients registered in practice of GP**				
≤ 1500		/	/	/
> 1500—≤ 3000		0.05 ± 0.04	0.05 ± 0.03	0.05 ± 0.04
> 3000—≤ 7000		0.05 ± 0.04	0.04 ± 0.04	0.02 ± 0.04
> 7000		0.07 ± 0.04	0.07 ± 0.04	0.04 ± 0.05
**Multidisciplinarity of the practice team**				
No		/	/	/
Yes		0.06 ± 0.03 *	0.05 ± 0.03	0.05 ± 0.03
**Availability of administrative support**				
No		/	/	/
Yes		0.04 ± 0.03	0.03 ± 0.03	0.03 ± 0.03
**Perception of adequate governmental Support**				
(Strongly) disagree		/		
Neutral		0.06 ± 0.03*	0.05 ± 0.03	0.05 ± 0.03
(Strongly) agree		0.15 ± 0.03***	0.17 ± 0.03***	0.17 ± 0.03***
**Covariance parameter estimate**				
Variance	0.09 ± 0.02***	0.08 ± 0.02***	0.08 ± 0.02***	0.08 ± 0.02***
Residual	0.40 ± 0.01***	0.40 ± 0.01***	0.40 ± 0.01***	0.40 ± 0.01***
**Model information**				
AIC	7118.2	6471.2	6290.3	6251.3
BIC	7123.1	6474.5	6294.3	6254.6
-2 log likelihood	7112.2	6467.2	6297.6	6247.3
Likelihood ratio test	/	(331.3) ***	(169.6) ***	(49.7) ***

^*^ p value < 0.05

^**^ p value < 0.01

^***^ *p value* < 0.001

In the three models, the "PE" score was not significantly influenced by the number of GPs per practice, the practices with patients with limited or low health literacy, the number of patients registered in the practice and the presence of administrative support.

## Discussion

Although many studies were carried out during the COVID-19 pandemic, they often focused on the curative aspects of the disease and were based in secondary care. Exploration on the preventive aspects used to tackle the COVID-19 pandemic, including educational activities are lacking. The international PRICOV-19 study rolled out in 38 countries provides a unique opportunity to describe PC during the COVID-19 pandemic. This paper examined the educational activities carried out in PCPs in order to identify characteristics more favourable to patient educational approaches in primary care.

Among the characteristics that seem supportive of engaging patient educational activities in PCPs, is the payment system when it is of the "capitation or mixed payment" type. This element may also have been reinforced during the health crisis, which particularly undermined a fee-for-service payment model. One study showed that fee-for-service payment is not very suitable in the event of a health crisis [25]. The number of face-to-face procedures performed by GPs was reduced during the COVID-19 pandemic and this may have put some professionals in financial difficulties [26]. Our results indicate that the capitation financing system may provide a better adaptation to ensure the solidity of the health system, as it does not depend on the volume of care actually provided and can therefore be more responsive to need. Moreover, this financing system has already been shown to result in a higher proportion of patients with recommended care and more favourable health outcomes [25]. Another study conducted during the COVID-19 pandemic provides quite same results where the fee-for-service system caused an increase in mortality among "non-COVID" patients and discontinuity of care through the closure of health centers [27]. Nevertheless, these studies do not indicate the precise reasons for the positive health outcomes related to capitation, nor whether they are directly related to educational activities. This demonstrates the value of further research on the subject in order to understand the mechanisms through which the funding model impacts.

The results also indicate another characteristic of PCPs that positively influences educational activities: the perception of average PCP with patients with chronic diseases. This is consistent with recommendations for these patients who were particularly at risk during the COVID-19 pandemic and for whom discontinuity of care needed to be limited [2, 10, 25]. The same relationship did not appear in this research for PC practices with patients with low or limited health literacy. This can be linked to another study which indicated the underestimation of this phenomenon by the health-care system as a whole [16].

Another favourable feature this research identifies is the perception of adequate government support. These results are in line with other studies that also suggest the importance of government support in providing stronger primary care and better health outcomes [25, 27]. However, these studies do not give a clear indication of how this support works. The emphasis on this type of support in the results thus points to the need to think about PC systemically with the involvement of all stakeholders, as also stated in the 2018 Astana Declaration [28].

However, the presence of a multidisciplinary team does not seem to be significantly related to educational activities. This finding is more surprising given the quality criteria for educational activities, which recommend involving the entire team, both medical and paramedical [29]. Indeed, this type of functioning makes it possible to have a complementary approach and a global patient vision, which is essential for this educational activity. However, it should be underlined that our results only show the descriptive aspect linked to the composition of a multidisciplinary practice rather than a multidisciplinary functioning. This observation certainly refers to the current lack of structuring of primary care, which does not allow for a definition of roles among the different disciplines, in particular for educational approaches [30].

### Strengths and limitations of the study

One strength of the study is its breadth, covering 38 countries, as well as its sample size, which is also large and makes its results more easily transferable to the wider region covered by the study.

One of the main limitations of the analyses presented in this paper is that the main focus of the study was not purely on educational activities. As a result, the variables studied were derived from the items present in the original study and not from specific items or validated questionnaires. Furthermore, we do not have in-depth details about the functioning or organisation of the educational activities by the professionals. This gives us a certainly reductive view of the possible scope of educational activities in primary care practices. However, the recent WHO report indicates that TPE includes information-sharing activities and that this activity constitutes one of the technical supports for TPE, in the service of shared decision-making [31]. This does not cover the most advanced forms of TPE. Despite this limitation, the PRICOV-19 study, with its large sample size, represented a unique opportunity to study the characteristics related to these educational activities.

Given the sampling methods used in PRICOV-19, which was based on self-selection, there may be a selection bias. However, this research has undergone a rigorous process of methodological development which is detailed in another article [19]. Given the sampling methods used in PRICOV-19, which are based on self-selection, it is possible that there is a selection bias. Although this research underwent a rigorous process of methodological development [19], limitations remain for this study because the data collection periods and recruitment strategies were adapted to each country and to the resources available during the PRICOV-19 pandemic. These limitations therefore affect the representativeness of the results.

Another limitation concerns the profile of study participants who are not fully representative of PC as a whole. Indeed, the responding practices have a higher proportion of large practices, which contradict the literature that shows a majority of monodisciplinary practice. However, it should be noted that multidisciplinary practice has been increasing in recent years in several European countries (e.g., Belgium, Switzerland and France) [32]. On the other hand, our results show a predominance of the capitation mode of payment, while the fee-for-service system remains in the majority in many countries [32]. These two characteristics may indicate a greater involvement of a more collective practice of care. This can be explained by the sampling method used, namely self-selection, as described in the article by Groenewegen et al. [33].

## Conclusions

As a key element in prevention, the patient educational approach was part of strategies implemented during the COVID-19 pandemic, including in primary care practices.

The results, based on the PRICOV study, indicate that these educational activities varied among the 38 countries investigated, but that there are factors associated with these practices that are more favourable, such as the presence of patients with chronic diseases, the capitation payment system or government support.

The model presented in this article is still incomplete and questions remain to better understand the functioning of PC practices in order to identify the brakes and levers for this educational approach in PC, not only in times of COVID-19 pandemic but also outside of pandemic times.

### Supplementary Information

**Additional file 1.** Residuals for the score "PE".

**Additional file 2. **Conditional Residuals for the score “PE”.

## Data Availability

The anonymized data is held at Ghent University and all raw data that could lead to the identification of the respondents were permanently removed. Reasonable request is required to access non-identifiable data by users who are external to the research teams involved. Access will be subject to a data transfer agreement and following approval from the principal investigator of the study.

## Abbreviations

COVID-19: Coronavirus Disease 2019
PRICOV-19: Quality and safety in PRImary care in times of COVid-19
PCP: Primary care practice
PE (score): Patient Education (score)
SD: Standard deviation
TPE: Therapeutic patient education
WHO: World Health Organization
PC: Primary care
WONCA: World Organization of Family Doctors
GP: General practitioner
LMM: Linear mixed model
ICC: Intraclass correlation coefficient
AIC: Akaike’s Information Criterion
BIC: Bayesian Information Criterion
SE: Standard error
