# Supplementary figures and images for: Characteristics of primary care practices associated with patient education during COVID-19: results of the cross-sectional PRICOV-19 study in 38 countries
Source: BMC Prim Care. 2024 Apr 18;24(Suppl 1):285. doi: 10.1186/s12875-024-02348-x (PMC11027213; doi:10.1186/s12875-024-02348-x)

**Supplementary materials**

## Additional file 1


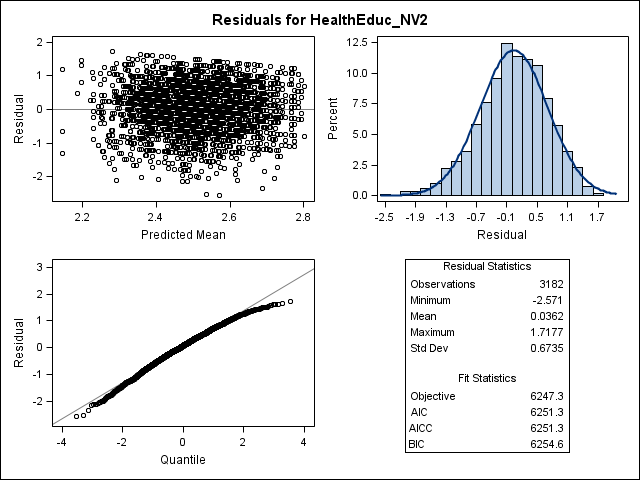

Supplement: Supplementary file 1 — Additional file 1. Residuals for the score "PE". [file 12875_2024_2348_MOESM1_ESM.docx]

**Additional file 2**


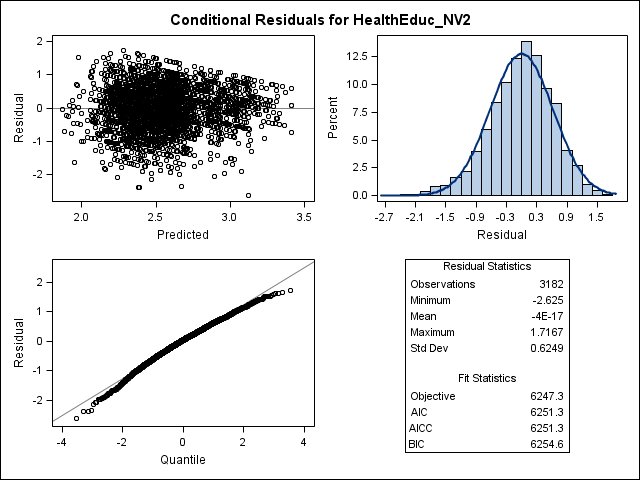

Supplement: Supplementary file 2 — Additional file 2. Conditional Residuals for the score “PE”. [file 12875_2024_2348_MOESM2_ESM.docx]
